# AI in clinical diagnostics: Is overreliance eroding clinical expertise?

**DOI:** 10.1371/journal.pdig.0000959

**Published:** 2025-08-04

**Authors:** Fawad A. Khan

**Affiliations:** 1 The International Center for Epilepsy at Ochsner, Ochsner Neuroscience Institute, Ochsner Health, New Orleans, Louisiana, United States of America; 2 The University of Queensland Medical School, Brisbane, Queensland, Australia; Shahid Beheshti University of Medical Sciences School of Dentistry, IRAN, ISLAMIC REPUBLIC OF

## Lessons in building core clinical reasoning skills

“Let’s see what the scans show,” I suggested while presenting a case of acute neurological changes to the attending physician during my neurology residency. Struggling to localize the deficits, I had turned prematurely to imaging—sidestepping the clinical reasoning and deductive process I was meant to cultivate. The attending gently reminded me that the clinical clues were right in front of me, encouraging reliance on clinical judgment over technological shortcuts.

In a prescient warning, my clinical professors during medical school cautioned against an overreliance on advanced neuroimaging, arguing that it would gradually erode the essential diagnostic skills honed through careful bedside assessment. They emphasized that mastering these foundational skills was crucial—not only to minimize unnecessary tests and costs but also to prevent misdiagnoses driven by incidental imaging findings that could obscure clinical judgment [1].

## The erosion of clinical expertise in the age of AI

In clinical neurology, localization remains the defining skill set—the hallmark of the discipline—guiding diagnosis and treatment through precise correlation of symptoms with neuroanatomy and neurophysiology. This skill needs constant honing, and no prolepsis of technological shortcuts will aid in its development.

Rather, it will erode the very abilities.

This essay calls for a critical reflection on the rapid integration of artificial intelligence (AI) into healthcare, urging both clinical educators and clinicians—the end users of these technologies—to reconsider the impact on our core diagnostic expertise.

## The unchecked expansion of machine learning in clinical diagnostics

Today, history repeats itself—this time under the banner of AI. As machine learning applications permeate nearly every facet of healthcare, we face a renewed paradigm shift in technology adoption, unfolding with even greater fervor. Despite the looming ethical and regulatory challenges surrounding AI in healthcare—such as data privacy concerns, biases from inadequately diverse training datasets, and insufficient oversight in commercialization—machine learning models are still eagerly embraced [2].

The allure of greater efficiency, improved diagnostic accuracy, reduced costs, expanded service capacity, and advanced predictive analytics continues to drive the rapid adoption [3]. In diagnostics, AI tools are streamlining workflows by enabling time savings and broadening capacity of diagnostic services using prescreening approaches. They are also advancing personalized medicine by analyzing large-scale datasets to extract meaningful insights, facilitate earlier interventions, and improve outcomes—particularly in vulnerable populations.

More recently, deep learning tools, fueled by increasingly sophisticated neural network architectures, have begun to permeate diagnostic workflows at an unprecedented pace. This transition is fostering a growing reliance on automated analysis—gradually overshadowing essential clinical skill sets, such as differential diagnosis, contextual interpretation, and critical appraisal of inherent variability in diagnostic data. While media narratives about AI “one day replacing physicians” remain speculative, a more germane concern is the quiet erosion of critical diagnostic skills that physicians spend years honing.

In clinical neurodiagnostics, I have witnessed the unsettling erosion of our core skills as technology rapidly takes center stage. The once-common sight of a perceptive neurologist, skilled in old-school methods, walking the hallways with a leather bag containing tuning forks, an optokinetic tape, and a reflex hammer is becoming an increasingly rare sight in modern clinical settings.

## AI in diagnostic electroencephalography—A Dicey path and the risks ahead

As an attending clinical neurophysiologist trained under the tutelage of renowned experts and highly skilled electroencephalographers, I spent countless hours reviewing raw electroencephalography (EEG) data, refining my pattern recognition skills. Today, I find myself confronted with the expansive capabilities of advanced artificial neural networks (ANNs) in EEG analysis, which offer unmatched speed and an exceptional ability to detect even the most subtle details and intricate patterns. While these advancements are undeniably impressive, my concerns remain unheeded—the art of EEG interpretation risks being marginalized, similar to how over-reliance on AI-powered navigation tools like Google Maps has dulled traditional wayfinding and spatial reasoning skills. On this note, I share two major concerns.

First, the performance of ANN models in EEG analysis relies heavily on the quality and diversity of training data. Many studies use data from restrictive sources and selective populations, often lacking real-world validation. Without improvements in this area, these tools could mislead clinicians and worsen existing healthcare disparities.

A common global challenge in hospital settings is the shortage of physicians trained in EEG interpretation, compounded by gaps in after-hours monitoring and limitations in reviewing the entirety of continuous, multi-day EEG recordings due to constrained personnel resources [4]. In response, there has been a growing—albeit pragmatic—reliance on health technology solutions. However, this approach carries significant caveats. It would be an ethical failure to miss a diagnosis of non-convulsive status epilepticus based on EEG data and delay treatment simply because the real-time ANN model assigned to this task failed to detect it. Unfortunately, documented instances of such failures have already appeared in the literature [5].

Second, the integration of these AI tools into EEG training during residency and fellowship programs presents a risky shortcut that can impact the formation of core competencies. A balanced approach is essential. Learning to effectively utilize AI tools and automated systems can improve efficiency, enhance speed, and potentially identify errors missed by human interpretation. However, this should be a secondary strategy, applied after a solid foundation has been built through traditional hands-on learning. Fundamental training must prioritize meaningful interaction with expert clinicians, allowing learners to make mistakes, receive constructive feedback, and strengthen their critical thinking. This apprentice-style learning—where skill development is closely paired with guided feedback—remains a cornerstone of medical training. Furthermore, experiential learning models are essential for the continued refinement and retention of core competencies.

## Safeguarding clinical expertise in the face of emerging tech integration

I am profoundly grateful to the educators in my fellowship training who limited the use of AI-assisted EEG analysis, ensuring we developed core skills through traditional methods. I continue to uphold these principles in our fellowship program. A survey study by Ahmad and colleagues on university learners found that using AI significantly diminishes human decision-making and fosters laziness [6]. Such shifts in mindset and attitudes could take root in current training programs, producing graduates who may go on to lead future training efforts. This could ultimately perpetuate a cycle that leads to the deterioration of expertise, creating an abyss of lost clinical skills.

The erosion of EEG interpretation skills is just one example within the broader spectrum of diagnostic fields in clinical medicine, where the meteoric rise in AI adoption is diminishing clinical human expertise. We must pause and critically assess the risks of this perilous journey. An insightful perspective on this challenge is offered by AI expert and author Mustafa Suleyman, who aptly states, “The promise of technology is that it improves lives, with benefits far outweighing the costs and downsides. However, this set of wicked choices has led to the savage inversion of that promise.” [7].

Our responsibility in this zeitgeist of rapid AI application scaling in clinical diagnostics is to ensure these technologies support—rather than supplant—the clinical judgment and acumen that defines medical practice.
